# Assessment of the prevalence of intestinal parasitic infections and associated habit and culture-related risk factors among primary schoolchildren in Debre Berhan town, Northeast Ethiopia

**DOI:** 10.1186/s12889-020-10148-y

**Published:** 2021-01-09

**Authors:** Gedamu Gebreamlak Hailu, Esubalew Tesfahun Ayele

**Affiliations:** Department of Public Health, Medicine and Health Science Institute, College of Health Science, Debre Berhan University, P. O. Box, 445 Debre Berhan, Ethiopia

**Keywords:** Intestinal parasite, Risk factors, Schoolchildren, Culture-related, Habit

## Abstract

**Background:**

Intestinal parasitic infections (IPIs) are still among the major public health issues in developing countries. Assessing the prevalence of IPIs and potential risk factors in different localities is essential to enhance control strategies. To date, no prevalence assessment study was conducted in Debre Berhan town. Therefore, the aim of this study was to assess the prevalence of IPIs and associated habit and culture-related risk factors among primary schoolchildren in Debre Berhan town, Northeast Ethiopia.

**Method:**

School based cross-sectional study was conducted from April to June 2017. A total of 645 children aged 6–15 years were selected from six primary schools in Debre Berhan town via a multistage random sampling technique. A structured questionnaire was used to collect data about sociodemographic and potential risk factor variables. Fresh stool samples were collected from each child and examined using direct smear and formal-ether concentration technique.

**Result:**

Among the 645 children participated in the study, 341 (52.9%) were infected by one or more intestinal parasites. Helminths (33.8%) were more prevalent than protozoa (20%). Double parasitic infection rate was 0.9%. The predominant parasites were *Ascaris lumbricoides* (22.6%), *Entamoeba histolytica/dispar/moshkovskii* (18.1%) and *Hymenolepis nana* (5.7%). Multivariable logistic regression analysis showed that age of child (6–9 years), family size (above 5), mother’s illiteracy and primary education, father’s illiteracy, urban-farmer father, manual-worker father, not washing hands before eating, unclean fingers, open defecation site (ODS) near residence, latrine type, cultural response to dropped food (cleaning and eating; ‘kiss and replace’), habit of playing with waste water, habit of playing with soil, habit of sucking fingers and habit of eating when playing were significantly associated with IPIs (*p*< 0.05). Likewise, age (6–9 years), mother’s illiteracy, urban-farmer father, not washing hands before eating, ODS near residence, tradition of cleaning and eating dropped food, habit of playing with soil, sucking fingers and eating when playing were identified as significant risk factors of *A. lumbricoides* infection.

**Conclusion:**

High prevalence of IPIs among the study participants demands improvement of environmental sanitation, personal hygiene, and health education regarding the potential habit and culture-related risk factors.

**Supplementary Information:**

The online version contains supplementary material available at 10.1186/s12889-020-10148-y.

## Background

Intestinal parasites contain helminths and protozoa that invade the gastrointestinal tract [1]. Infections caused by intestinal parasites have been known to cause huge health problems of mankind without restrictions [1–3]. The soil transmitted helminths (STHs) alone affect above 2.5 billion people worldwide [4]. Besides, the protozoa *Entamoeba histolytica* and *Giardia lamblia* infect about half a billion individuals worldwide [1–3, 5]. Intestinal parasites are mainly transmitted via the fecal-oral route. The majority of these parasites are orally acquired, while some species such as hookworm and *Strongyloides stercoralis* infect by skin penetration [2, 6].

Intestinal parasitic infections (IPIs) are widespread in developing countries. Particularly in Sub-Saharan Africa, IPIs are the major public health problems [7–9]. Intestinal parasitic infections affect people of all ages; however, school-aged children (6–15 years) are the most affected and important risk groups [8–10]. The effect of infection is also debilitating for the children, because it occurs during a period of rapid metabolism, intense physical growth and learning, impairing their physical and intellectual growth; causing much suffering and death [11].

Distributions of IPIs often are linked to lack of access to clean water, poor sanitation and poor hygiene; hence, they largely affect children living in resource limited areas where poverty prevails [7]. However, the distribution and prevalence of various IPIs differs across endemic countries and among various localities within a country because of several social, environmental and climatic factors. Thus, studies on the prevalence of IPIs and potential risk factors in different localities are essential not only to identify high risk communities but also to enhance control strategies.

Several studies have been conducted in different countries worldwide and diverse prevalence rates of IPIs (10–88.2%) were reported among schoolchildren [12–17]. Likewise, a number of studies have been conducted in different localities in Ethiopia and high prevalence rates of IPIs (26.9–83.8%) were reported among schoolchildren [18–28]. However, there are still several localities for which epidemiological information is not available. Most of the previous studies in Ethiopia were conducted at rural and semi-urban settings [18–25]; while fewer studies were done in urban areas [26–28]. The present study area is one of the urban areas in Ethiopia, where prevalence assessment studies have not been conducted yet.

Several previous studies done in Ethiopia and elsewhere have also investigated the risk factors of IPIs and we are already clued-up about the associations of different sociodemographic, hygiene and environmental sanitation factors with the presence of IPIs [12–20, 22–27]. Whereas, various habit and cultural**-**related risk factors in different localities, which may aggravate the fecal-oral route of intestinal parasites transmission, are still overlooked. In line with this, systematically investigating the potential risk factors for IPIs considering the local and cultural practices in different localities is vital to enhance control strategies [29]. Therefore, the aim of this study was to assess the prevalence of IPIs and potential risk factors including the habit and culture-related practices among primary schoolchildren in Debre Berhan town, Northeast Ethiopia.

## Methods

### Study design and study area

School based cross-sectional study was conducted from April to June 2017 among primary schoolchildren in Debre Berhan town, Northeast Ethiopia. Debre Berhan is located about 120 km far from the capital city of the country, Addis Ababa. The altitude of the area is 2780 m above sea level. The average annual temperature for day and night is 17.8°c and 8.83°c, respectively. Debre Berhan is the capital of North Shoa zone and one of the fast growing cities in Ethiopia. It has a total population of 113, 693 [30]. There are 26 primary schools in Debre Berhan that enroll about fourteen thousand school-aged children.

### Study population

The study population consisted of school-aged children (6–15 years) attending class from grade 1 to grade 8 in selected primary schools of Debre Berhan town.

### Sample size and sampling technique

The sample size was determined by using a statistical formula [n = (Z^2^_α/2_ P (1-P)/ d^2^] where P (prevalence of IPIs in the area), d (at 5% marginal error) and standard score (Z) at 95% confidence interval (CI). Since there was no similar study conducted previously in the area, 50% prevalence rate of IPIs was taken. Considering the 1.6 design effect (because of a multistage sampling technique), the sample size determined for the study was 614. In order to minimize errors caused by possible dropout, estimated non-response rate of 5% was added giving a final sample size of 645 study participants.

A multi-stage random sampling technique was applied. The 26 primary schools in the town were organized in five school-clusters by the town’s education office based on relative proximity. The school-clusters were considered ideal groupings for this study. Thus, firstly four of the five school-clusters (primary sampling units) were selected by a lottery method. In the second stage, six schools were randomly selected from the four school-clusters with probability proportional to size. Finally, 645 school-aged children were randomly selected from the six primary schools based on proportional allotment to total number of children in each school and grade level.

### Data collection

#### Sociodemographic data and associated risk factors

A structured questionnaire was used to collect relevant information from each schoolchild. The questionnaire was developed based on known risk factors and assumed habit and culture-related practices of children in the study area and elsewhere in Ethiopia. It was first prepared in English and translated into Amharic (native language). A pre-test was conducted using 5% of the sample size on a non-study sample population of primary schoolchildren in a nearby town. Finally, selected students for this study were interviewed to obtain information about the necessary sociodemographic, environmental sanitation, hygiene, habit and culture-related factors. At the same time, factors such as cleanness of fingers were assessed through direct observation using a checklist set in the questionnaire (Additional file 1). The interview was carried out by trained primary school teachers from a different school, with the assistance of field research supervisors.

#### Stool specimen collection and examination

Stool specimen examination was performed using two parasitological techniques (direct smear and formal-ether concentration) based on the World Health Organization (WHO) standard specimen processing and diagnosis guideline [31]. Following the interview, each schoolchild was asked to bring fecal specimens using properly labeled clean plastic stool collecting cup with an applicator stick/spoon for adding about 5 g of fresh stool. Four experienced laboratory technologists were employed for the study. The direct smear examination was performed on fresh stool samples mixed with 0.85% saline, at the field laboratories within less than 30 min of collection by two of the laboratory technologists. All developmental stages of the parasites (trophozoite, cyst, egg, larvae and adult) were recorded.

The formal**-**ether stool concentration technique was performed on preserved stool. Two other laboratory technologists added about 3 g part of the fresh stool specimens delivered by each child into a disposable 15 ml screw sealed centrifuge tube labelled with the child’s unique code, mixed with 3 ml (10%) formalin for preservation and transported to the Medical Microbiology Laboratory of Debre Berhan University. Then, all preserved specimens were processed using formal-ether stool concentration techniques as indicated in the WHO guidelines [31] by the laboratory technologists. Similarly, all developmental stages of the parasites (cyst, egg, larvae and adult) were recorded.

### Quality control

All laboratory procedures and sample handling were done by trained laboratory technologists based on standards [31]. Reagents were checked using preserved specimens known negative, and positive for parasite ova and cyst. Laboratory technologists worked in pair, crosschecking each stool specimen blindly one after the other both in the field and the university. To make all procedures effective, training was given to all data collectors and supervisors. Orientation was given to children. On spot checks, re-interviewing and heedful examination of completed questionnaires and quality of recordings were done through daily supervisions.

### Data analysis

The data were analyzed using SPSS version 16.0. Descriptive statistics were computed. Logistic regression analysis was used to verify the possible associations of potential risk factors with the presence of IPIs. In the modeling process, a bivariate logistic regression analysis was first done for each of the potential risk factor variables. Those independent variables with a *p*-value < 0.2 in the bivariate analysis were purposely selected and entered into the final multivariable logistic regression model [32]. All variables supposed to have confounding effect were crosschecked independently. Multicollinearity among independent variables was checked using a correlation matrix computed along with the final model. In the final model, a significant association was declared at a *p*-value < 0.05. And finally, the results were presented in texts and tables. Adjusted odds ratios (AOR) with the corresponding 95% confidence interval were used to explain the strength of associations between the dependent and independent variables.

### Operational definitions

**Clean and eat dropped food:** cultural response to the food dropped when eating. It involves picking, cleaning and eating the food so as to show respect and get forgiveness of superpower.

**Culture-related risk factors:** factors related to the cultural practice of a given society and the children, which are assumed to facilitate the fecal-oral transmission of intestinal parasites.

**Habit-related risk factors:** factors related to the play habit, ritual practice and life style of children, which are assumed to facilitate the fecal-oral transmission of intestinal parasites.

**Intestinal parasite(s) positive:** a stool sample which contains a parasite ova, cyst or trophozoite in either of the two microbiological techniques.

**Kiss and replace dropped food:** cultural response to the food dropped when eating. It involves picking the food soon, kissing it and putting it back in a safe place. It is done instead of cleaning and eating dropped food so as to show respect and get forgiveness of superpower.

**Manual work:** labor activities that are usually non-professional and contextually low paid.

**Open defecation site (ODS) near residence:** the presence of fields, bushes, ditches or streets at the child’s residence, where people are frequently defecating in the open.

**School-aged children:** children from age 6 to 15 years.

**School-cluster:** a group of primary schools in the town that are officially organized by the town’s education office for managerial and logistics purpose based on relative proximity.

### Ethical consideration

Ethical clearance was obtained from the Institutional Review Board of Debre Berhan University, Institute of Medicine and Health Science (Reference number–DBUMF 053–009). It was further communicated to the Zone’s and the Town’s Health and Education Departments/Offices. Verbal consent was obtained from the schools’ principals and a written informed consent was obtained from parents/guardians on behalf of all child participants. Finally, in collaboration with the North Shoa Zone Health Department, infected children were treated without cost.

## Result

### Socio demographic characteristics of the study participants

A total of 645 children aged 6–15 years participated in this study on voluntary basis. Among these children, 337 (52.2%) were girls and the remaining 308 (47.8%) boys. Three hundred thirty-six (52.1%) and 309 (47.9%) children were from grades 1 to 4 and grades 5 to 8, respectively. About half of the children (51.9%) came from family sizes of 5 and below. About 28% of the children’s fathers and close to 33% of the children’s mothers were illiterate (Table 1).

**Table 1:** Sociodemographic characteristics of the study participant primary schoolchildren and their family in Debre Berhan town, Northeast Ethiopia, 2017

Variables	Category	Frequency	Percent
Sex	Boys	308	47.8
	Girls	337	52.2
Age of child in years	6–9	204	31.6
	10–12	231	35.8
	13–15	210	32.6
Birth order of child	First	212	32.9
	Non-First	433	67.1
Living with birth parents	No	86	13.3
	Yes	559	86.7
Family size	2–5	335	51.9
	6–9	310	48.1
Grade level of child	1–4	336	52.1
	5–8	309	47.9
Father education	Illiterate	182	28.2
	Primary education	184	28.5
	Junior education	81	12.6
	Secondary education	80	12.4
	Diploma and above	118	18.3
Mother education	Illiterate	212	32.9
	Primary education	116	18.0
	Junior education	108	16.7
	Secondary education	70	10.9
	Diploma and above	139	21.6
Father occupation	Farmer (urban farming)	102	15.8
	Manual worker	171	26.5
	Trader/ petit-trade	187	29.0
	Employee	185	28.7

### Prevalence of intestinal parasites among the study participants

Among the 645 children participated in the study, 341 (52.9%) were infected by one or more parasitic organism (Table 2). The prevalence of intestinal parasitic infection was 54.5 and 51.3% among male and female children, respectively. The prevalence of helminths and protozoa was 33.8 and 20.0%, respectively. The rate of double parasitic infections was 0.9% (Table 2). Seven parasitic organisms were identified from the study participants. Among these parasites, *A. lumbricoides* was the most prevalent 146 (22.6%) followed by *E. histolytica/dispar/moshkovskii* 117 (18.1%), *H. nana* 37 (5.7%), hookworm 25 (3.9%) and *G. lamblia* 12 (1.9%) (Table 2).

**Table 2:** Prevalence of intestinal parasites among primary schoolchildren in Debre Berhan town, Northeast Ethiopia, 2017 (*N*=645)

Parasitic species	Total No (%)	Boys No (%)	Girls No (%)	*P*-value
All protozoa	129 (20.0)	67 (21.7)	62 (18.4)	0.288
Entamoeba spp.	117 (18.1)	62 (20.1)	55 (16.3)	0.211
*Giardia lamblia*	12 (1.9)	5 (1.6)	7 (2.1)	0.668
All helminths	218 (33.8)	106 (34.4)	112 (33.2)	0.751
*Ascaris lumbricoides*	146 (22.6)	74 (24.0)	72 (21.4)	0.420
*Hymenolepis nana*	37 (5.7)	16 (5.2)	21 (6.2)	0.570
Hookworm	25 (3.9)	10 (3.2)	15 (4.4)	0.425
*Strongyloides stercoralis*	6 (0.9)	3 (1.0)	3 (0.9)	0.912
*Teania saginata*	4 (0.6)	3 (1.0)	1 (0.3)	0.285
All double parasite infection	6 (0.9)	5 (1.6)	1 (0.3)	0.088
*A. lumbricoides +* hookworm	3 (0.45)	2 (0.6)	1 (0.3)	0.518
*A. lumbricoides + H.nana*	1 (0.15)	1 (0.3)	0 (0.0)	0.317
*A. lumbricoides +* Entamoeba spp.	1 (0.15)	1 (0.3)	0 (0.0)	0.317
Entamoeba spp. *+ G. lamblia*	1 (0.15)	1 (0.3)	0 (0.0)	0.317
Total positives (At least one species)	341 (52.9)	168 (54.5)	173 (51.3)	0.414
Total negatives	304 (47.1)	140 (45.5)	164 (48.7)	0.414

### Multivariable analysis of potential risk factors associated with intestinal parasitic infections

Multivariable logistic regression analysis was used to identify the most significant risk factors for IPIs among primary schoolchildren in Debre Berhan town (Table 3). Data about twenty-nine potential risk factor variables within three categories (i.e. sociodemographic, hygiene and environmental sanitation, and habit and culture-related risk factors) were gathered from the study participants (Additional file 2). In the modeling process, a bivariate logistic regression analysis was first done for each of the risk factors. Those independent variables with a *p*-value < 0.2 in the bivariate analysis were selected for multivariable analysis [32]. In view of that procedure, eighteen variables were selected for final analysis, whereas eleven variables including the sex of child, soap use for hand washing and shoes wearing habit were not selected (Table 3).

**Table 3:** Bivariate and multivariable logistic regression analysis of potential risk factors associated with IPIs among primary schoolchildren in Debre Berhan town, Northeast Ethiopia, 2017 (*N*=645)

Variables	Category	Intestinal Parasite		OR (95% C.I)	
		Positive	Negative	Crude	Adjusted
Sociodemographic factors					
Age (years)	6–9	127	77	1.34 (0.90, 1.98)	3.10 (1.31, 7.31)*
	10–12	98	133	0.60 (0.41, 0.87)*	0.65 (0.28, 1.49)
	13–15	116	94	1	1
Sex of child	Boys	168	140	1.14 (0.84, 1.55)	–
	Girls	173	164	1	
Birth order	Non- First	244	189	1.53 (1.10, 2.13)*	0.82 (0.39, 1.69)
	First	97	115	1	1
Living with birth parents	No	49	37	1.21 (0.77, 1.91)	–
	Yes	292	267	1	
Family size	≥ 6	237	73	7.21 (5.08, 10.23)*	6.84 (3.31,14.12)**
	2–5	104	231	1	1
Father education	Illiterate	119	63	7.80 (4.51, 13.50)*	3.85 (1.40,10.60)**
	Primary education	132	52	10.49 (6.01,18.30)*	2.47 (0.78, 7.75)
	Junior education	36	45	3.30 (1.76, 6.22)*	2.54 (0.76, 8.49)
	Secondary education	31	49	2.61 (1.38, 4.96)*	2.28 (0.70, 7.41)
	Diploma and above	23	95	1	1
Mother education	Illiterate	158	54	24.19 (13.03,44.90)*	5.81 (1.99,16.92)**
	Primary education	68	48	11.71 (6.11, 22.45)*	4.56 (1.52,13.66)**
	Junior education	69	39	14.63 (7.53, 28.42)*	3.03 (0.90, 10.18)
	Secondary education	31	39	6.57 (3.22, 13.42)*	2.86 (0.84, 9.65)
	Diploma and above	15	124	1	1
Father occupation	Urban farming	86	16	10.66 (5.77, 19.72)*	8.16 (2.55,26.12)**
	Manual worker	110	61	3.58 (2.31, 5.54)*	3.27 (1.28, 8.37)*
	Trader/petit-trade	83	104	1.58 (1.04, 2.41)*	2.10 (0.88, 5.03)
	Employed	62	123	1	1
Family IPs infection history	Yes	144	125	1.05 (0.77, 1.43)	–
	No	197	179	1	
Hygiene and environmental sanitation factors					
Hand wash before eating	Not always	240	114	3.96 (2.85, 5.50)*	8.23 (3.94,17.15)**
	Always	101	190	1	1
Hand wash after defecation	Not always	227	178	1.41 (1.02, 1.94)*	0.92 (0.44, 1.93)
	Always	114	126	1	1
Finger cleanness	Not clean	228	85	5.20 (3.71, 7.28)*	2.66 (1.30, 5.46)**
	Clean	113	219	1	1
Source of water	Stream	33	5	7.50 (2.88, 19.53)*	2.64 (0.37, 18.68)
	Public tap	80	40	2.27 (1.49, 3.46)	1.49 (0.57, 3.90)
	Piped water	228	259	1	1
Type of latrine	PLWOS	75	17	7.39 (4.21, 12.99)*	5.43 (1.47, 20.09)*
	PLWS-shared	115	34	5.67 (3.68, 8.73)*	2.79 (1.18, 6.60)*
	PLWS-private	151	253	1	1
ODS near residence	Yes	229	85	5.27 (3.76, 7.38)*	3.58 (1.77, 7.25)**
	No	112	219	1	1
Soap use for hand wash	Not regular	286	264	0.79 (0.51, 1.22)	–
	Regular	55	40	1	
Disposal of waste in home yard	Yes	210	177	1.15 (0.84, 1.58)	–
	No	131	127	1	
Presence of pets in home	Yes	235	204	1.09 (0.78, 1.51)	–
	No	106	100	1	
Habit and culture-related factors					
Habit of playing with waste water	Yes	218	88	4.35 (3.12, 6.06)*	7.99 (3.60,17.71)**
	No	123	216	1	1
Habit of playing with soil	Yes	247	90	6.25 (4.44, 8.80)*	6.25 (2.75,14.16)**
	No	94	214	1	1
Habit of eating while playing	Yes	257	125	4.38 (3.13, 6.13)*	2.80 (1.23, 6.38)*
	No	84	179	1	1
Cultural response to dropped food	Cleaning and eating	117	26	8.72 (5.03, 15.10)*	11.42 (3.82,34.11)**
	Kiss and replace	176	185	1.84 (1.23, 2.76)*	4.08 (1.71, 9.74)**
	Leaving it there	48	93	1	1
Habit of sucking fingers/pen-caps	Yes	198	90	3.29 (2.37, 4.57)*	4.05 (1.92, 8.55)**
	No	143	214	1	1
Habit of eating on the way to school	Yes	315	204	5.94 (3.73, 9.47)*	2.39 (0.88, 6.45)
	No	26	100	1	1
Habit of using grass as toothpick	Yes	207	167	1.27 (0.93, 1.73)	–
	No	134	137	1	
Habit of playing barefoot	Yes	78	58	1.26 (0.86, 1.84)	–
	No	263	246	1	
Eating raw vegetables	Rarely	141	122	1.05 (0.77, 1.44)	–
	Not at all	200	182	1	
Eating raw meat	Yes	91	92	0.84 (0.60, 1.18)	–
	No	250	212	1	
Shoes wearing habit	Not regular	20	13	1.40 (0.68, 2.85)	–
	Regular	321	291	1	

Note**:** *= significant at *p*-value < 0.05, **= significant at *p*-value < 0.01, *PLWOS* Pit-latrine without slab, *PLWS* Pit-latrine with slab, *ODS* Open defecation site, *OR* Odds ratio

The multivariable logistic regression analysis showed that the sociodemographic factors age of child 6–9 years (AOR=3.10; 95% CI, 1.31–7.31), family size above 5 (AOR=6.84; 95% CI, 3.31–14.12), illiterate father (AOR=3.85; 95% CI, 1.40–10.60), illiterate mother (AOR=5.81; 95% CI, 1.99–16.92), mother with primary education (AOR=4.56; 95% CI, 1.52–13.66), urban-farmer father (AOR=8.16; 95% CI, 2.55–26.12) and manual-worker father (AOR=3.27; 95% CI, 1.28–8.37) were significantly associated with IPIs (Table 3). Likewise, the hygiene and environmental sanitation factors not washing hands before eating (AOR=8.23; 95% CI, 3.94–17.15), unclean fingers (AOR=2.66; 95% CI, 1.30–5.46), pit-latrine without slab (AOR=5.43; 95% CI, 1.47–20.09), shared pit-latrine with slab (AOR=2.79; 95% CI, 1.18–6.60) and open defecation site (ODS) near residence (AOR=3.58; 95% CI, 1.77–7.25) were identified as significant risk factors of IPIs (Table 3).

Above all, the habit and culture-related practices of children such as cultural response to dropped food (cleaning and eating dropped food (AOR=11.42; 95% CI, 3.82**–**34.11); ‘kiss and replace dropped food’ (AOR=4.08; 95% CI, 1.71**–**9.74)), habit of playing with waste water (AOR=7.99; 95% CI, 3.60**–**17.71), habit of playing with soil (AOR=6.25; 95% CI, 2.75**–**14.16), habit of sucking fingers/pen-caps (AOR=4.05; 95% CI, 1.92**–**8.55) and habit of eating when playing (AOR=2.80; 95% CI, 1.23**–**6.38) were identified as significant risk factors of IPIs (Table 3).

### Potential risk factors associated with *Ascaris lumbricoides* infection

*Ascaris lumbricoides* was the leading intestinal parasite identified from the study participants (Table 2). Multivariable logistic regression analysis showed that age of child 6–9 years, mother’s illiteracy, urban-farmer father, not washing hands before eating, ODS near residence, tradition of cleaning and eating dropped food, habit of playing with soil, habit of sucking fingers/pen-caps and eating when playing were significantly associated with *A. lumbricoides* infection (Table 4).

**Table 4:** Bivariate and multivariable logistic regression analysis of potential risk factors associated with Ascaris infection among primary schoolchildren in Debre Berhan town, Northeast Ethiopia, 2017 *(N*=645)

Variables	Category	*A. lumbricoides*		OR (95% C.I)	
		Positive	Negative	Crude	Adjusted
Sociodemographic factors					
Age (years)	6–9	58	146	1.64 (1.04, 2.59)*	2.61 (1.45, 4.70)**
	10–12	47	184	1.05 (0.66, 1.68)	1.55 (0.87, 2.77)
	13–15	41	169	1	1
Sex of child	Boys	74	234	1.16 (0.81, 1.68)	–
	Girls	72	265	1	
Birth order	Non- First	103	330	1.23 (0.82, 1.83)	–
	First	43	169	1	
Living with birth parents	No	22	64	1.21 (0.71, 2.04)	–
	Yes	124	435	1	
Family size	≥ 6	99	211	2.88 (1.95, 4.25)**	1.24 (0.76, 2.01)
	2–5	47	288	1	1
Father education	Illiterate	58	124	4.55 (2.27, 9.11)**	1.55 (0.65, 3.69)
	Primary education	53	131	3.94 (1.96, 7.91)**	0.98 (0.39, 2.45)
	Junior education	12	69	1.69 (0.71, 4.05)	0.78 (0.27, 2.26)
	Secondary education	12	68	1.72 (0.72, 4.11)	1.33 (0.48, 3.71)
	Diploma and above	11	107	1	1
Mother education	Illiterate	71	141	8.25 (3.82, 17.79)**	2.95 (1.15, 7.58)*
	Primary education	30	86	5.71 (2.50, 13.05)**	2.66 (1.00, 7.08)
	Junior education	28	80	5.73 (2.49, 13.19)**	2.02 (0.72, 5.71)
	Secondary education	9	61	2.42 (0.89, 6.57)	1.40 (0.44, 4.44)
	Diploma and above	8	131	1	1
Father occupation	Urban farming	41	61	4.30 (2.41, 7.67)**	2.20 (1.10, 4.42)*
	Manual worker	44	127	2.22 (1.29, 3.82)**	0.93 (0.48, 1.80)
	Trader/petit-trade	36	151	1.53 (0.88, 2.66)	1.25 (0.63, 2.46)
	Employed	25	160	1	1
Family IPs infection history	Yes	65	204	1.16 (0.80, 1.68)	–
	No	81	295	1	
Hygiene and environmental sanitation factors					
Hand wash before eating	Not always	101	253	2.18 (1.47, 3.23)**	2.05 (1.24, 3.39)**
	Always	45	246	1	1
Hand wash after defecation	Not always	91	314	0.98 (0.67, 1.43)	–
	Always	55	185	1	
Finger cleanness	Not clean	89	224	1.92 (1.32, 2.79)**	0.76 (0.47, 1.23)
	Clean	57	275	1	1
Source of water	Stream	14	24	2.29 (1.14, 4.58)*	1.57 (0.55, 4.48)
	Public tap	33	87	1.49 (0.94, 2.35)	1.37 (0.73, 2.58)
	Piped water	99	388	1	1
Type of latrine	PLWOS	28	64	2.16 (1.29, 3.62)**	1.20 (0.53, 2.74)
	PLWS-shared	50	99	2.50 (1.63, 3.83)**	1.47 (0.86, 2.52)
	PLWS-private	68	336	1	1
ODS near residence	Yes	102	212	3.14 (2.11, 4.66)**	1.74 (1.05, 2.85)*
	No	44	287	1	1
Soap use for hand wash	Not regular	126	424	1.11 (0.66, 1.90)	–
	Regular	20	75	1	
Disposal of waste in home yard	Yes	93	294	1.22 (0.84, 1.79)	–
	No	53	205	1	
Presence of pets in home	Yes	103	336	1.16 (0.78, 1.74)	–
	No	43	163	1	
Habit and culture-related factors					
Habit of playing with waste water	Yes	90	216	2.11 (1.44, 3.07)**	1.51 (0.94, 2.45)
	No	56	283	1	1
Habit of playing with soil	Yes	122	215	6.72 (4.19, 10.77)**	3.91 (2.19, 7.00)**
	No	24	284	1	1
Habit of eating while playing	Yes	119	263	3.96 (2.51, 6.22)**	1.82 (1.07, 3.30)*
	No	27	236	1	1
Cultural response to dropped food	Cleaning and eating	52	91	3.46 (1.93, 6.19)**	2.58 (1.25, 5.34)*
	Kiss and replace	74	287	1.56 (0.91, 2.67)	1.72 (0.90, 3.28)
	Leaving it there	20	121	1	1
Habit of sucking fingers/pen-caps	Yes	92	196	2.63 (1.80, 3.86)**	1.82 (1.11, 2.96)*
	No	54	303	1	1
Habit of eating on the way to school	Yes	137	382	4.66 (2.30, 9.44)**	1.63 (0.70, 3.84)
	No	9	117	1	1
Habit of using grass as toothpick	Yes	92	282	1.31 (0.90, 1.92)	–
	No	54	217	1	
Habit of playing barefoot	Yes	27	109	0.81 (0.51, 1.30)	–
	No	119	390	1	
Eating raw vegetables	Rarely	54	209	0.81 (0.56, 1.19)	–
	Not at all	92	290	1	
Eating raw meat	Yes	33	150	0.68 (0.44, 1.50)	–
	No	113	349	1	
Shoes wearing habit	Not regular	5	28	0.60 (0.23, 1.57)	–
	Regular	141	471	1	

*Note****:*** *= significant at *p*-value < 0.05, **= significant at *p*-value < 0.01, *PLWOS* Pit-latrine without slab, *PLWS* Pit-latrine with slab, *ODS* Open defecation site, *OR* Odds ratio

## Discussion

Epidemiological studies on the prevalence of intestinal parasitic infections (IPIs) and potential risk factors in different localities are vital to update the risk of communities under consideration and to enhance control strategies. Accordingly, this study tried to assess the prevalence of IPIs and potential risk factors including the habit and culture-related practices among primary schoolchildren in Debre Berhan town.

In this study, seven types of intestinal parasites were detected among the participants with an overall prevalence of 52.9%. This prevalence was consistent with the study which was conducted in Bahir Dar town, Ethiopia [28]. It was also a bit higher compared with 26.9–44.2% reported in different parts of Ethiopia [18–20, 27]. However, the present finding was lower compared with 77.5–88.2% reported in some rural and semi-urban areas of Ethiopia [21–25] and other countries [16, 17]. These reported disparities in prevalence of IPIs among different studies might be explained by differences in climatic conditions, parasitological methods used, urban and rural settings, family education and career, level of environmental sanitation and hygiene, habit and culture-related practice of the study subjects, and previous control efforts.

Ascaris lumbricoides was the most prevalent species (22.6%). This prevalence was lower than the study conducted in Tilili [18], Delgi [23] and Chencha [24]. However, this figure was very high compared with other similar studies in Ethiopia [19–22, 25–28]. As for other STHs, the prevalence of hookworm (3.9%) in this study was lower than the school-based study conducted in Bahirdar town [28]. In contrast to several previous studies in Ethiopia, no *Trichuris trichiura* infection was identified among the present study participants [18–28]. Nonetheless, this finding was consistent with previous studies elsewhere [12, 13, 16]. The development of whipworm eggs into infective stages is affected by hot and cold surface temperatures [33]. Altitude beyond 1800–2500 m was also shown to be not suitable for *T. trichiura* [33, 34]. Although our finding alone is not enough to infer nonexistence of whipworm in the present study area, the extremely low temperature and the high altitude of the area might inhibit incidence of the worm to a negligible rate.

*Hymenolepis nana* was the second most prevalent (5.7%) parasitic helminths in this study. This prevalence was quite higher compared with several previous studies conducted in different parts of Ethiopia [18–20, 24–26]. However, it was lower than the study which was conducted in Gondar, Ethiopia [27]. *Strongyloides stercoralis* infection rate was lower compared with other studies in Ethiopia [21, 25]. *Taenia saginata* infection rate was also found to be lower than other studies [15, 25].

Regarding protozoal infection, the overall prevalence (20.0%) was lower than the school-based study conducted in Dagi [22] and Delgi [23]. In agreement with other similar studies in Ethiopia, the prevalence of E. histolytica/dispar/moshkovskii (18.1%) was very high [22–24, 26], but this finding was not consistent with the study conducted in Gondar town [27]. On the other hand, the prevalence of *G. lamblia* (1.9%) was remarkably lower compared with several previous studies conducted in different parts of Ethiopia [20, 22–27] and abroad [5, 16].

Combating intestinal parasites needs a holistic approach that integrates chemotherapeutic intervention with tailored health education programs [2, 29]. Children living in various localities are innocently exposed to diverse risk factors for IPIs. The systematic investigation of potential risk factors for IPIs considering the local and cultural situation in different localities is vital to enhance control strategies.

Accordingly, the present study showed that the sociodemographic variables age of child, family size, parent education and father occupation were strongly associated with IPIs. Age of child 6–9 years and family size above 5 were significantly associated with IPIs, which was consistent with the results previously reported [26]. This might be due to lower age children are relatively less aware than the older age children to be exposed to various risk factors for IPIs. As well, the latter might be due to the relatively limited resource and inadequate cares often given to children in a large family compared with the children in a small family. Father’s illiteracy, mother’s illiteracy and primary education were significantly associated with presence of IPIs in children, which was consistent with the results previously reported elsewhere [13, 24, 35]. Urban-farming and manual work careers of fathers were also significantly associated with presence of IPIs in children. This finding was consistent with other study in Dagi [22].

Results also showed that the hygiene and environmental sanitation factors including hand hygiene, latrine type and open defecation site (ODS) were strongly associated with IPIs. Not washing hands before eating and unclean fingers were significantly associated with IPIs, which was consistent with the results previously reported in other studies [20, 23, 26–28]. Suggesting unwashed hands and dirty fingers are liable to keep parasite ova/cyst thus can increase risk of infection. Pit-latrine without slab and shared pit-latrine with slab were significantly associated with IPIs. This finding was consistent with other study [24]. Open defecation sites often preserve parasite cyst/ova [2]. This study evaluated the presence of ODS near the child’s residence in witness of the respondent child. The finding also showed the children who witnessed the presence of ODS near their residence were more likely to acquire IPIs than the children who witnessed no ODS. This result was in line with other studies [15, 24].

Above all, this study attempted to assess the habit and culture-related risk factors of IPIs. To our knowledge, the risk factors were not studied previously in Ethiopia. The present finding showed the likelihood of being infected by IPIs was increased by about 8 and 6 folds among children who had the habit of playing with waste water and with soil, respectively, compared with the children who had not the respective habit. Likewise, children who had the habit of sucking their fingers/pen-caps had about 4 folds higher chances of being infected with IPIs compared with the children who had not the habit. Although we did not find empirical studies done yet, these findings might be explained by the fact that IPIs are prevalent wherever transmitting agents contaminated with parasitic ova/cyst are enormous [2].

As well, the children who had the habit of eating while playing had about 3 folds higher chances of being infected with IPIs compared with the children who had not the habit. Children in the study area have the custom of eating snacks in late afternoon while playing outside home. This habit might increase direct contamination of the food they eat or their hands with soil or waste, and eventually may cause them swallow the parasitic ova/cyst.

Cultural response to dropped food was the foremost risk factor that showed strong association with the presence of IPIs in this study. The chance of being infected with IPIs was increased by about 11 and 4 folds among children who had the practice of cleaning and eating dropped food and those who had the practice of ‘kissing and replacing’ dropped food, respectively, compared with the children who were freed from these cultural practices. The possible explanation for this association might be due to the contamination of dropped food with soil or potential wastes that contain parasite ova/cyst and the increased chance of ingesting the infective agents.

### Limitations of the study

This study has some limitations. First, the study was limited to only schoolchildren in Debre Berhan town. Including schoolchildren outside the town would have been better to get a bigger picture of the prevalence of IPIs as well as the associated habit and culture-related risk factors in North Shoa Zone. Second, the study used a single fecal sample which may underestimate the prevalence of IPIs. Third, the study did not apply a more sensitive diagnostic method that could distinguish the morphologically identical distinct species of the genus *Entamoeba* that were reported as *E. histolytica/dispar/moshkovskii*; and additional method for *S. stercoralis*. However, to our knowledge this study is the first of its type to look for the associated habit and culture-related risk factors for IPIs among schoolchildren in Ethiopia.

## Conclusions

The study revealed a high prevalence of intestinal parasitic infections (IPIs) among primary school children in Debre Berhan town. Both helminths and protozoa infections are common in the schoolchildren. Multiple risk factors such as lower ages, large family size, parents’ illiteracy, urban-farmer father, manual-worker father, poor hand washing habit, unclean fingers, pit-latrine without slab, shared pit-latrine with slab, open defecation site near residence, habit of eating while playing, habit of sucking fingers, habit of plying with soil and waste water, cleaning and eating, and ‘kissing and replacing’ dropped food were strongly associated with IPIs. High prevalence of IPIs in the schoolchildren demands enhanced intervention actions. Therefore, in planning and implementing intervention actions all stakeholders should give attention to improve environmental sanitation, raise awareness concerning personal hygiene and the potential habit and culture-related risk factors.

## Supplementary Information

**Additional file 1.** English version of questionnaire

**Additional file 2.** Raw data

## Data Availability

The data analyzed during this study are included in this article. The row dataset is also attached as an Additional file 2, and it is available from the corresponding author on reasonable request.

## Abbreviations

AOR: Adjusted Odds Ratio
CI: Confidence Interval;
OR: Odds Ratio
IPIs: Intestinal Parasitic Infections
ODS: Open Defecating Site
SPSS: Statistical Package for Social Science
STHs: Soil Transmitted Helminths
WHO: World Health Organization.
