# Supplementary material for: Assessment of the prevalence of intestinal parasitic infections and associated habit and culture-related risk factors among primary schoolchildren in Debre Berhan town, Northeast Ethiopia
Source: BMC Public Health. 2021 Jan 9;21:112. doi: 10.1186/s12889-020-10148-y (PMC7797111; doi:10.1186/s12889-020-10148-y)
Supplement: Supplementary file 1 — Additional file 1. English version of questionnaire [file 12889_2020_10148_MOESM1_ESM.docx]

## Questionnaires /English version

**I. Structured questionnaires/ for interview/**

**Part one**: Students school record

1. Name of the School ________________________________School Code_________________

2. Child Code __________________________________________________________________

3. Grade _______________ Section _____________

4. Date of interview _________ ________ __________

**Part two: Demographic Factors**

1. Age_________________

2. Sex Male Female

1. Sometimes
2. Rarely
3. Never
4. State if any other……………………………………..

1. Sometimes
2. Rarely
3. Never
4. State if any other……………………………………..

3. Birth order _________ (as first, second- third, etc.)

4. Do you live with your birth parents? Yes No

1. Sometimes
2. Rarely
3. Never
4. State if any other……………………………………..

1. Sometimes
2. Rarely
3. Never
4. State if any other……………………………………..

5. Family Size (total) _________ (number of siblings at home/ brothers and sisters only) _____

1. Sometimes
2. Rarely
3. Never
4. State if any other……………………………………..

1. Sometimes
2. Rarely
3. Never
4. State if any other……………………………………..

6. Residence: Urban Kebeles Rural Kebeles

**Part three: Socio-economic Factors**

1. Is your father educated?

a. Yes b. No

2. If yes, what is his educational status?

a. Primary education (write on the respected grade level) ________________

b. Junior-education (write on the respected grade level) ________________

c. Secondary education (write on the respected grade level) ________________

d. College Diploma ___________

e. University Degree___________

f. Above (mention) ___________

3. Is your mother educated?

a. Yes b. No

4. If yes, what is her educational status?

a. Primary education (write on the respected grade level) ________________

b. Junior-education (write on the respected grade level) ________________

c. Secondary education (write on the respected grade level) ________________

d. College Diploma ___________

e. University Degree___________

f. Above (mention) ___________

5. What is the occupation of your father?

a. Farmer b. Government-employed c. NGO-employed d. Merchant

e. Petty trade (Small and micro) f. Unemployed g. Labour/day labour h. Self-employed

i. State if any others_________________________________________________

6. What is the occupation of your mother?

a. Farmer b. Government-employed c. NGO-employed d. Merchant

e. Petty trade (Small and micro) f. Unemployed g. Labour/day labour h. Self-employed

i. House wife j. State if any others____________________________________

7. How much is the monthly income of your family in Ethiopian Birr? _______________ ETB

8. Have you ever been infected with any of the intestinal parasites? Yes No

9. Is there any one in your family who was infected with any of the intestinal parasites? Yes No

**Part four: Habits/ Behavioural Factors**

1. Do you wash hands before you eat food? Yes No

2. If yes, how often do you wash hands before you eat food?

1. Regularly
2. Sometimes
3. Rarely
4. State if any other……………………………………..

3. If yes, what do you use to wash your hands?

1. Water only
2. Water and ash
3. Water and soap
4. It depends(not consistent)

4. Do you wash hands after using toilet? Yes No

5. If yes, how often do you wash hands after you use toilet?

1. Regularly
2. Sometimes
3. Rarely
4. State if any other……………………………………..

6. If yes, what do you use to wash your hands?

1. Water only
2. Water and ash
3. Water and soap
4. It depends(not consistent)

7. Do you eat raw vegetables? Yes No

8. If yes, how often do you eat raw vegetables?

1. Usually
2. Sometimes
3. Rarely
4. State if any other……………………………………..

9. Do you wash the raw vegetables you eat? Yes No

10. If yes, how often do you wash the raw vegetables?

1. Regularly
2. Sometimes
3. Rarely
4. State if any other……………………………………..

11. Do you eat raw meat? Yes No

1. Sometimes
2. Rarely
3. Never
4. State if any other……………………………………..

1. Sometimes
2. Rarely
3. Never
4. State if any other……………………………………..

12. Do you wear shoes? Yes No

1. Sometimes
2. Rarely
3. Never
4. State if any other……………………………………..

1. Sometimes
2. Rarely
3. Never
4. State if any other……………………………………..

13. If yes, how often do you wear shoes?

1. Regularly
2. Some times
3. Rarely
4. State if any other……………………………………..

14. Do you have a habit of taking off shoes while playing with friends? Yes No

1. Sometimes
2. Rarely
3. Never
4. State if any other……………………………………..

1. Sometimes
2. Rarely
3. Never
4. State if any other……………………………………..

15. If yes, how often do you take off your shoes while playing?

1. Regularly
2. Sometimes
3. Rarely
4. State if any other…………………………………….

16. Do you have a habit of playing with soil? Yes No

1. Sometimes
2. Rarely
3. Never
4. State if any other……………………………………..

1. Sometimes
2. Rarely
3. Never
4. State if any other……………………………………..

17. Do you have a habit of playing with waste water?

Yes No

1. Sometimes
2. Rarely
3. Never
4. State if any other……………………………………..

1. Sometimes
2. Rarely
3. Never
4. State if any other……………………………………..

18. Do you have a habit of eating food while playing out with friends? Yes No

1. Sometimes
2. Rarely
3. Never
4. State if any other……………………………………..

1. Sometimes
2. Rarely
3. Never
4. State if any other……………………………………..

19. If yes, how often do you eat food while playing out with friends?

1. Regularly
2. Sometimes
3. Rarely
4. State if any other_____________________________

20. Have you ever defecating open in the field or bush? Yes No

1. Sometimes
2. Rarely
3. Never
4. State if any other……………………………………..

1. Sometimes
2. Rarely
3. Never
4. State if any other……………………………………..

21. If yes, how often do you defecating open in the field or bush?

1. Regularly
2. Sometimes
3. Rarely
4. State if any other______________________________

22. Is there any one among your friends who defecate open around the field/bush /?

Yes No

1. Sometimes
2. Rarely
3. Never
4. State if any other……………………………………..

1. Sometimes
2. Rarely
3. Never
4. State if any other……………………………………..

23. If yes how often they do?

1. Regularly
2. Sometimes
3. Rarely
4. State if any other_____________________________

24. Have you ever observed people around your residence open defecating? Yes No

1. Sometimes
2. Rarely
3. Never
4. State if any other……………………………………..

1. Sometimes
2. Rarely
3. Never
4. State if any other……………………………………..

25. If yes how often they do?

1. Regularly
2. Sometimes
3. Rarely
4. State if any other_____________________________

26. Do you have the habit of cutting your fingernails with your teeth? Yes No

1. Sometimes
2. Rarely
3. Never
4. State if any other……………………………………..

1. Sometimes
2. Rarely
3. Never
4. State if any other……………………………………..

27. If yes how often you do so?

1. Regularly
2. Sometimes
3. Rarely
4. State if any other_____________________________

28. Do you have the habit of sucking your fingers pen/cups/ and the like with your teeth or inserting in to your mouth? Yes No

1. Sometimes
2. Rarely
3. Never
4. State if any other……………………………………..

1. Sometimes
2. Rarely
3. Never
4. State if any other……………………………………..

29. If yes how often you do?

1. Regularly
2. Sometimes
3. Rarely
4. State if any other_____________________________

30. Have you ever buy yourself or your friends and eat foods that are sold around your school?

Yes No

1. Sometimes
2. Rarely
3. Never
4. State if any other……………………………………..

1. Sometimes
2. Rarely
3. Never
4. State if any other……………………………………..

31. If the food you are eating dropped down from your hand while you are eating, what are you going to do?

1. I will clean it and eat
2. I will pick and through it, then continue eating the rest
3. I will leave it there and continue eating the rest
4. State if any other_______

**Part five: Environmental Factors**

1. From where do you get water for drinking and cooking?

1. Private tab
2. Public tab
3. Rain water
4. Stream

2. How do you use the water for drinking?

1. Treating with chemicals
2. Boiling
3. Without any treatment
4. State if any other________________________________________

3. Is latrine available? Yes No

1. Sometimes
2. Rarely
3. Never
4. State if any other……………………………………..

1. Sometimes
2. Rarely
3. Never
4. State if any other……………………………………..

4. If yes, what type of latrine is it? Traditional pit latrine Slit trench latrine

1. Sometimes
2. Rarely
3. Never
4. State if any other……………………………………..

1. Sometimes
2. Rarely
3. Never
4. State if any other……………………………………..

Conventional improved pit latrine VIP latrine Pour-flush latrine

1. Sometimes
2. Rarely
3. Never
4. State if any other……………………………………..

1. Sometimes
2. Rarely
3. Never
4. State if any other……………………………………..

1. Sometimes
2. Rarely
3. Never
4. State if any other……………………………………..

***NB:*** *For clear understanding, simply ask children to tell what the latrine they use looks like, the materials from which it is made of, the availability of attached water source to clean, the presence of wall, roof, type and nature of sits etc. And fill the information on the space next.*

***____________________________________________________________________________________________________________________________________________________________________________________________________________________________________________________________________________________________________________________________________________________________________________________________________________________________________________________________________________________***

5. If yes is it? Private Common

1. Sometimes
2. Rarely
3. Never
4. State if any other……………………………………..

1. Sometimes
2. Rarely
3. Never
4. State if any other……………………………………..

6. If common, how many people use it? (Put the response in number) ________________

7. Is there any water available in the latrine? Yes No

1. Sometimes
2. Rarely
3. Never
4. State if any other……………………………………..

1. Sometimes
2. Rarely
3. Never
4. State if any other……………………………………..

8. If available what type of water is it?

a. A tab attached to it b. Water stored in a portable container

9. If No latrine, where do you defecate and dispose faeces?

1. Near the streams
2. Open field
3. Using pits and disposes to the streams
4. State if any other ____________________________________________________

**Part six: Personal and communal hygiene**

I. Personal hygiene (Direct observation)

1. Hand hygiene

1. Are finger nails trimmed? Yes No
2. Sometimes
3. Rarely
4. Never
5. State if any other……………………………………..

1. Sometimes
2. Rarely
3. Never
4. State if any other……………………………………..

1. Sometimes
2. Rarely
3. Never
4. State if any other……………………………………..

1. Is there any dirt seen inside fingernails? Yes No
2. Sometimes
3. Rarely
4. Never
5. State if any other……………………………………..

1. Is there any dirt seen on the palms? Yes No
2. Sometimes
3. Rarely
4. Never
5. State if any other……………………………………..

1. Sometimes
2. Rarely
3. Never
4. State if any other……………………………………..

1. Is there any dirt seen on any of the fingers? Yes No
2. Sometimes
3. Rarely
4. Never
5. State if any other……………………………………..

1. Sometimes
2. Rarely
3. Never
4. State if any other……………………………………..

2. Full personal hygiene

1. Is there any dirt seen on the faces? Yes No
2. Sometimes
3. Rarely
4. Never
5. State if any other……………………………………..

1. Sometimes
2. Rarely
3. Never
4. State if any other……………………………………..

1. Is there any dirt seen on the hairs? Yes No
2. Sometimes
3. Rarely
4. Never
5. State if any other……………………………………..

1. Sometimes
2. Rarely
3. Never
4. State if any other……………………………………..

1. Is there any dirt seen on the clothes? Yes No
2. Sometimes
3. Rarely
4. Never
5. State if any other……………………………………..

1. Sometimes
2. Rarely
3. Never
4. State if any other……………………………………..

1. Is there any dirt seen on the foot? Yes No
2. Sometimes
3. Rarely
4. Never
5. State if any other……………………………………..

1. Sometimes
2. Rarely
3. Never
4. State if any other……………………………………..

1. Write your judgement of the overall personal hygiene of the child (as, good or bad) on the space given next.

--------------------------------------------------------------------------------------

II. Communal hygiene (interviewing)

1. Are there pets in your home (cats, dogs, etc)? Yes No

1. Sometimes
2. Rarely
3. Never
4. State if any other……………………………………..

1. Sometimes
2. Rarely
3. Never
4. State if any other……………………………………..

2. Do the garbage collected from your home stay indisposed for longer in your yard?

Yes No

1. Sometimes
2. Rarely
3. Never
4. State if any other……………………………………..

1. Sometimes
2. Rarely
3. Never
4. State if any other……………………………………..

3. Is there a place around your residence where people usually open defecate?

Yes No

1. Sometimes
2. Rarely
3. Never
4. State if any other……………………………………..

1. Sometimes
2. Rarely
3. Never
4. State if any other……………………………………..

4. Is there a place on your way to school where people usually open defecate?

Yes No

1. Sometimes
2. Rarely
3. Never
4. State if any other……………………………………..

1. Sometimes
2. Rarely
3. Never
4. State if any other……………………………………..

5. Is there a place around your residence where people usually dispose the household wastes?

Yes No

1. Sometimes
2. Rarely
3. Never
4. State if any other……………………………………..

1. Sometimes
2. Rarely
3. Never
4. State if any other……………………………………..

6. Is there a place on your way to school where people usually dispose the household wastes?

Yes No

1. Sometimes
2. Rarely
3. Never
4. State if any other……………………………………..

1. Sometimes
2. Rarely
3. Never
4. State if any other……………………………………..
